# Guidewire-assisted endoscopic mucosal resection with over-the-scope clips for a cecal submucosal tumor

**DOI:** 10.1055/a-2607-8386

**Published:** 2025-06-26

**Authors:** Toru Kuwano, Shunji Shimaoka, Hirotake Kusumoto, Hideyuki Kishita, Tsutomu Sakiyama, Yukiko Baba, Saori Furukawa

**Affiliations:** 1 73609 Gastroenterology, Nanpuh Hospital, Kagoshima, Japan

A 48-year-old woman was referred to our hospital for examination and treatment of a cecal submucosal tumor. Colonoscopy was performed for pretreatment screening. The lesion in the cecum measured 5 mm, and the overlying mucosa was intact ( Fig. 1 ). Conventional endoscopic mucosal resection (EMR) or endoscopic submucosal dissection (ESD) would have carried a high risk of perforation and obscured the vertical margin 1 . We therefore decided to perform EMR with over-the-scope (OTS) clips (EMRO).

**Fig. 1:**
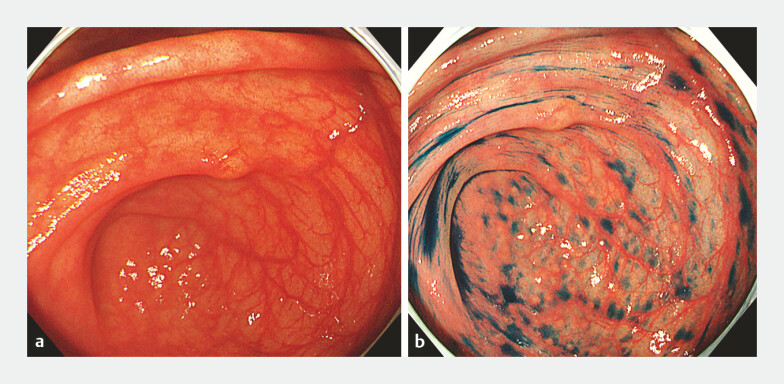
Colonoscopic images during pretreatment screening showing: **a** a submucosal tumor measuring 5 mm located in the cecum; **b** the tumor border revealed on indigo carmine staining.

During the colonoscopy for pretreatment screening, insertion was time-consuming and the patient experienced pain owing to postoperative adhesions, meaning delivery of the OTS clip might have been difficult. We therefore planned to perform guidewire-assisted EMRO (GA-EMRO). First, we inserted the scope into the cecum, and the proximal side of the target lesion was marked using a snare tip. We then placed the guidewire, and carefully withdrew the scope ( Fig. 2 ). We attached an OTS clip to the tip of the scope and safely reinserted it into the cecum with guidewire assistance. The lesion, including the marking, was suctioned into the cap. The OTS clip was successfully deployed, and the lesion was resected with a snare without complications ( Fig. 3 and Fig. 4 ; Video 1 ). Histopathologic examination confirmed a CD34-positive spindle tumor ( Fig. 5 ), with immunohistochemical analysis showing that the spindle cells were positive for CD34 and negative for α-SMA, desmin, c-kit, S-100, and factor XIIIa.

**Fig. 2:**
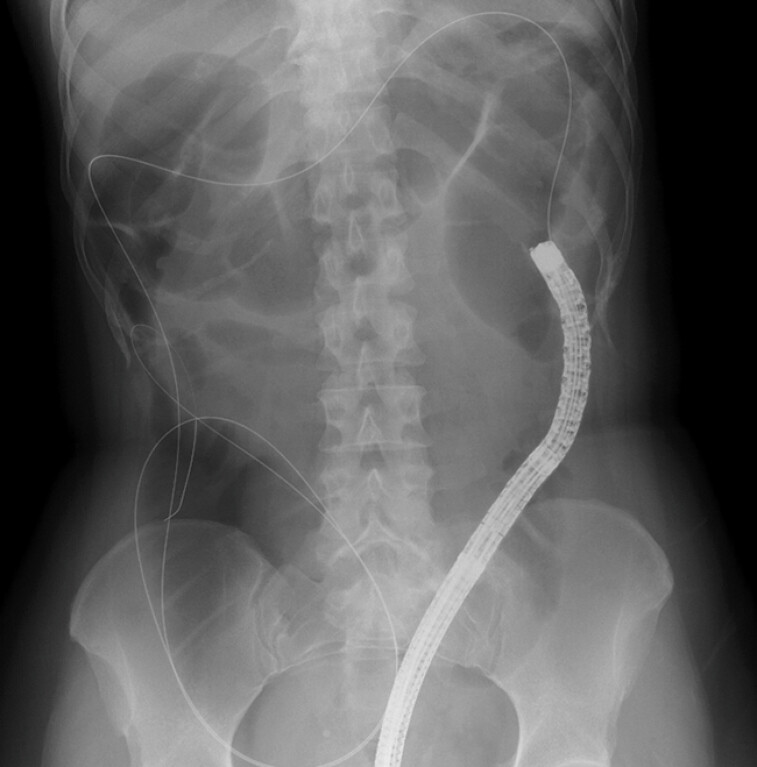
Radiographic image showing the inserted guidewire at the tip of the scope, which was confirmed, on imaging, as remaining in the cecum as the scope was carefully withdrawn.

**Fig. 3:**
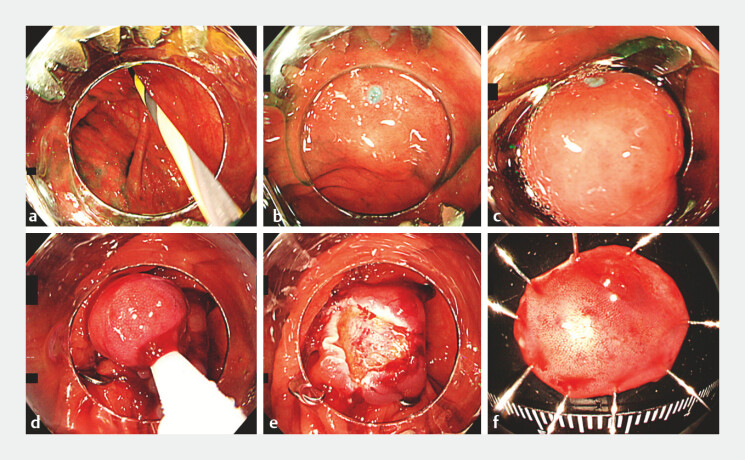
Images of guidewire-assisted endoscopic mucosal resection with over-the-scope (OTS) clips (GA-EMRO) being performed for a submucosal tumor in the cecum showing: **a** the scope with the mounted OTS clip being carefully inserted following the guidewire; **b** the cecal lesion and the previously placed mark; **c** the lesion and the marking suctioned into the cap; **d** the successfully deployed OTS clip and the lesion resected with a snare; **e** the defect post-resection; **f** the en bloc resected specimen.

**Fig. 4:**
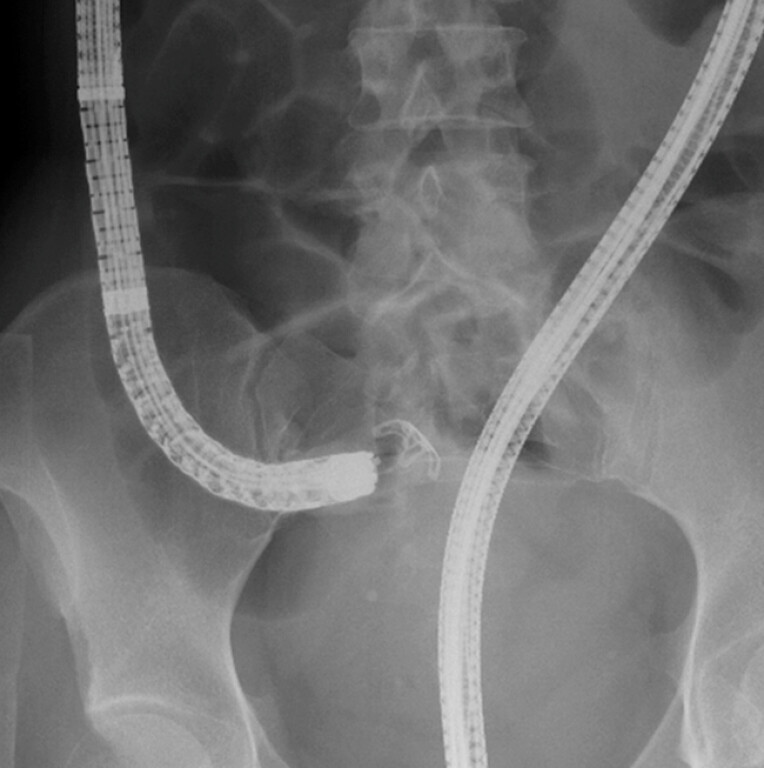
Radiographic image showing the deployment of the over-the-scope clip.

**Fig. 5:**
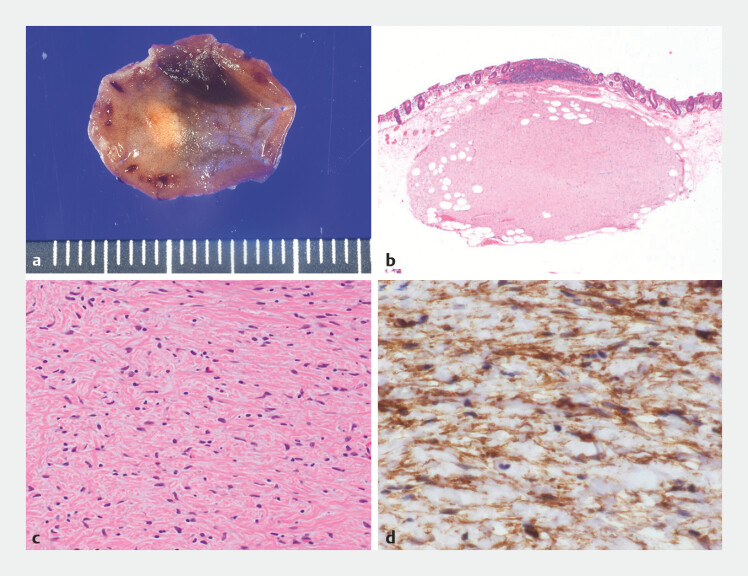
Images of the pathologic examination showing: **a** the macroscopic appearance; **b, c** on histopathologic appearance after hematoxylin and eosin (H&E) staining: **b** a clearly demarcated tumor in the submucosal layer (low power magnification); **c** the presence of spindle cells (high power magnification); **d** positivity for CD34 on immunohistochemical staining.

Guidewire-assisted endoscopic mucosal resection with over-the scope clips (GA-EMRO) is performed for a submucosal tumor in the cecum.Video 1

A limitation of OTS clips is that they restrict the endoscopic view when attached to the tip of the scope, and delivering the OTS clip may also be problematic 2 3 . EMRO has been reported to be a safe technique for the treatment of duodenal neuroendocrine tumors 4 . We performed GA-EMRO for a submucosal tumor in the cecum. Guidewire-assisted delivery is a valuable and safe method for OTS clip placement in the cecum, and the GA-EMRO technique can facilitate OTS clip treatment of lesions in the proximal large intestine.

Endoscopy_UCTN_Code_TTT_1AQ_2AD_3AF

## Notes

Endoscopy E-Videos https://eref.thieme.de/e-videos
*E-Videos* is an open access online section of the journal *Endoscopy* , reporting on interesting cases and new techniques in gastroenterological endoscopy. All papers include a high-quality video and are published with a Creative Commons CC-BY license. Endoscopy E-Videos qualify for HINARI discounts and waivers and eligibility is automatically checked during the submission process. We grant 100% waivers to articles whose corresponding authors are based in Group A countries and 50% waivers to those who are based in Group B countries as classified by Research4Life (see: https://www.research4life.org/access/eligibility/ ). This section has its own submission website at https://mc.manuscriptcentral.com/e-videos .
